# Extracellular Vesicle-Based Communication May Contribute to the Co-Evolution of Cancer Stem Cells and Cancer-Associated Fibroblasts in Anti-Cancer Therapy

**DOI:** 10.3390/cancers12082324

**Published:** 2020-08-18

**Authors:** Gábor Valcz, Edit I. Buzás, Anna Sebestyén, Tibor Krenács, Zoltán Szállási, Péter Igaz, Béla Molnár

**Affiliations:** 1 2nd Department of Internal Medicine and MTA-SE Molecular Medicine Research Group, 1051 Budapest, Hungary; igaz.peter@med.semmelweis-univ.hu (P.I.); molnar.bela1@med.semmelweis-univ.hu (B.M.); 2 Department of Genetics, Cell- and Immunobiology, Semmelweis University, 1089 Budapest, Hungary; buzas.edit@med.semmelweis-univ.hu; 3 MTA-SE Immune-Proteogenomics Extracellular Vesicle Research Group, Hungarian Academy of Sciences, 1089 Budapest, Hungary; 4 Hungarian Center of Excellence Molecular Medicine-Semmelweis University Extracellular Vesicle Research Group, 1085 Budapest, Hungary; 5 1st Department of Pathology and Experimental Cancer Research, Semmelweis University, 1085 Budapest, Hungary; sebestyen.anna@med.semmelweis-univ.hu (A.S.); krenacs.tibor@med.semmelweis-univ.hu (T.K.); 6 Computational Health Informatics Program (CHIP), Boston Children’s Hospital and Harvard Medical School, Boston, MA 02115, USA; Zoltan.Szallasi@childrens.harvard.edu

**Keywords:** extracellular vesicles, cancer stem cell, cancer cell-fibroblast interaction, therapy resistance

## Abstract

Analogously to the natural selective forces in ecosystems, therapies impose selective pressure on cancer cells within tumors. Some tumor cells can adapt to this stress and are able to form resistant subpopulations, parallel with enrichment of cancer stem cell properties in the residual tumor masses. However, these therapy-resistant cells are unlikely to be sufficient for the fast tumor repopulation and regrowth by themselves. The dynamic and coordinated plasticity of residual tumor cells is essential both for the conversion of their regulatory network and for the stromal microenvironment to produce cancer supporting signals. In this nursing tissue “niche”, cancer-associated fibroblasts are known to play crucial roles in developing therapy resistance and survival of residual stem-like cells. As paracrine messengers, extracellular vesicles carrying a wide range of signaling molecules with oncogenic potential, can support the escape of some tumor cells from their deadly fate. Here, we briefly overview how extracellular vesicle signaling between fibroblasts and cancer cells including cancer progenitor/stem cells may contribute to the progression, therapy resistance and recurrence of malignant tumors.

## 1. Introduction

Darwin’s natural selection theory in ecosystems shares many similarities with the evolution of tumors considering that cancer cells can adapt to their ever-changing microenvironment affected also by therapeutic interventions [1,2]. The genetic and epigenetic changes cooperatively produce intratumoral heterogeneity, which may promote the development of competitive cancer cell phenotypes [3]. Of the selection advantages provided by intratumoral heterogeneity, increased therapeutic resistance represents one of the greatest challenges in the clinical management of cancer patients [4]. Even after a seemingly successful therapy, a small population of tumor cells may survive as a minimal residual disease, which frequently remains hidden even to sensitive radiological imaging or histological examination [5]. Cells of minimal residual disease may develop through positive selection of newly mutated, resistant clones (clonal evolution hypothesis). Alternatively, they may originate from resistant cancer stem cells (CSCs), which lie at to the top of the tumor hierarchy (cancer stem cell hypothesis) [6,7,8]. These two models are not mutually exclusive and both may contribute to intratumoral heterogeneity [9]. 

The similarities in developmental stages, receptor expression patterns and self-renewal capacities of normal stem cells and CSCs raise the possibility that malignantly transformed normal stem cells may be a source of CSCs during tumor initiation [10,11]. In tumors that follow the CSC model, cancer cells can exist in different states of differentiation with a CSC population of relatively high replicative potential (producing highly proliferating transit-amplifying cells), which can contribute to intratumoral heterogeneity in the parent tumor [9,12,13,14]. In solid tumors, CSCs are identified by specific markers or marker combinations (e.g., CD44 and CD24 in breast cancer; CD133, CD166 and aldehyde dehydrogenase in colorectal cancer; CD133, CD44, aldehyde dehydrogenase and epithelial cell adhesion molecule in pancreatic cancer) [15]. Parallel testing of marker expression and CSC function (e.g., sphere formation ability) can be useful with some limitations (e.g., not all tumor-initiating cells show CSC markers, and some CSC marker positive cells may not behave as CSCs) [16]. The genotypic and phenotypic heterogeneity in the CSC population may serve as a critical reservoir for evolutionary tumor progression that enables adaptation to therapies [9,17,18]. In general, CSCs may be inherently resistant to apoptosis induction by cytotoxic agents and to radiation therapy (RT) as compared to the less undifferentiated bulk cancer cells [19]. As a consequence, even when the tumor mass is reduced, CSCs enriched in mutations, may accumulate within the remainder of the tumor [20,21]. These results strongly suggest that CSCs are responsible for the therapy-resistant populations of residual disease. For the survival of minimal residual disease, it may be critical that some cancer cell fates do not fit into the unidirectional hierarchical CSC model, and show dedifferentiation towards the progenitor or stem cell state (“cancer cell plasticity”) [13]. A high degree of cancer cell plasticity can be found both in epithelial-to-mesenchymal transition (EMT) and in proneural-to-mesenchymal transition where mesenchymal cells may acquire CSC-like characteristics [22,23]. 

**Figure 1:**
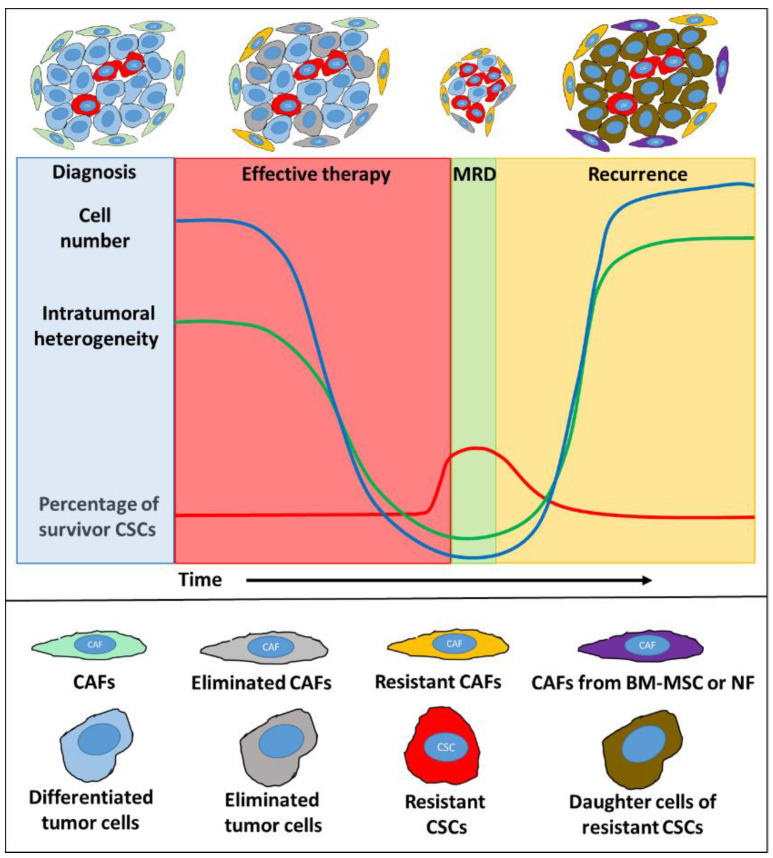
Summary of presumptive changes in carcinoma-associated fibroblast (CAF) populations, intratumoral heterogeneity, numbers of cancer cells, and percentages of survivor cancer stem cells (CSCs) during therapy and recurrence. In the course of chemotherapy, the majority of cancer cells (blue) are eliminated (grey). In parallel, the percentage of CSCs (red) may increase in minimal residual disease (MRD) compared to the gross tumor mass. During recurrence, daughter cells of the resistant CSCs can repopulate (brown) the tumor. The original CAF population (green) is partially eliminated (grey) by the therapy. Resistant (yellow) and newly formed CAFs may have dedifferentiated from normal fibroblasts (NFs) and from bone-marrow-derived mesenchymal stem cells (BM-MSCs) (purple).

Cancer cells can interfere with the physiological organization and the initial tumor-suppressing effect of the adjacent microenvironment [24]. As a response, the aberrant stroma composed of varying proportions of reactive cell types may produce extracellular matrix, enzymes and soluble growth factors to generate a functionally complex cancer supporting milieu, as described in Paget’s ‘seed-and-soil’ hypothesis [25]. During tumor evolution, malignant cells can also force plasticity in their microenvironment through instructive paracrine signaling. Locally acting regulators may induce stromal heterogeneity even around the same lesion due to the varying sensitivity of target cells. This could be the result of heterogeneous receptor expression/sensitivity, inflammatory or metabolic signals, acidic pH shift, as well as oxygen and nutrient supply [4,26,27,28,29]. Of these factors, hypoxia seems to be of particular importance in maintaining stem cell properties (including undifferentiated and slowly-dividing states and maintenance of the stem cell niche) [30,31,32]. Cancer cells respond to these regional differences. Therefore, stromal heterogeneity may deliver locally variable selection forces and promote intratumoral heterogeneity [20]. Moreover, radiation therapy (RT) and chemotherapy can also impose additional pressure on the tumor microenvironment resulting in phenotypically and metabolically modified stromal cell subpopulations [33,34,35,36,37]. Anti-cancer therapies can induce alarm signals in tumor-stroma interactions, which can select out cancer cells of CSC phenotypes, and activate cells in the adjacent stroma. Results of *in vitro* and *in vivo* studies (see examples below) suggest that a molecular symbiosis may evolve between cancer cells and stroma with potentially unfavorable clinical behavior. Among the non-immune cell types of the tumor stroma, carcinoma-associated fibroblasts (CAFs) are the most abundant ones in almost all solid tumors [38]. CAFs are heterogeneous and they consist of diverse subpopulations of cells found at different stages of their maturation [34,39,40]. Contrary to the tumor-suppressing role of fibroblasts and myofibroblasts in normal tissues, CAFs can provide functional and structural support to malignant cells [41,42,43]. Figure 1 summarizes the presumed changes in (i) cell number, (ii) intratumoral heterogeneity [44], (iii) presence of CSCs (in breast and colorectal cancer models [45,46,47]), and (iv) in CAF population (in breast cancer models [34,48] and hypothetically, based on a gastric cancer model [49]) during and after the therapy.

Cancer cells and the adjacent stroma may communicate through diverse pathways, such as soluble regulators, tunneling nanotubes, tumor microtubules and/or connexin channel-mediated direct intercellular connections [50,51,52,53]. One of the most prominent discoveries of recent years has been the recognition that an extracellular vesicle (EV)-based information exchange takes place between cells in nature. A continuous reciprocal EV-mediated communication also occurs between cancer cells and adjacent stroma cells. EVs are membrane-enclosed vesicles, which carry unique molecular signatures of various regulatory ligands, coding and non-coding nucleic acids, proteins, energy-rich metabolites as well as whole organelles [54]. Several hallmarks of cancer, including altered proliferative properties, growth suppression, cell death, energy metabolism, tumor microenvironment and acquired genomic instability are known to be affected by EV-associated oncogenic signals [55]. EVs also contribute to the development of a resistant residual disease and to the de-differentiation of cancer cells towards CSC phenotypes. The EV-based paracrine communication can ensure evolutionary benefits for both the cancer cells and fibroblasts. Thus, EVs appear to be important factors of the co-evolution of these cells. EVs modify the therapeutic sensitivity of cells by different mechanisms, such as (i) by sequestering and releasing drugs from the cells, (ii) conveying drug transporter molecules, (iii) capturing monoclonal antibodies of target therapy as well as (iv) by affecting post-transcriptional regulation and receptor-ligand interactions [56]. 

## 2. Tumor-Stroma Co-Evolution Requires CAF Survival, Recruitment, and Differentiation 

### 2.1. Therapy-Resistant Subpopulations of Carcinoma-Associated Fibroblasts 

As we described above, the evolving cancer cells may be crucially dependent on their specific, reactive microenvironment. This symbiotic relationship between cancer cells and CAFs may get intensified partially in response to a treatment-related evolutionary pressure [24,42]. For some CAF subpopulations, RT and chemotherapy may act as activators of tumor supportive functions [48,57,58,59]. The clinical relevance of the tumor-supporting phenotype of CAFs can be assessed by using specific resistance markers. Newly identified surface markers such as CD10 and the G protein-coupled receptor 77, may reflect resistance against docetaxel and cisplatin in double-positive CAFs. Importantly, these CAFs generate a nursing microenvironment (i.e., survival niche) around aldehyde dehydrogenase 1-positive CSCs under chemotherapeutic pressure in breast and lung cancer patients [34].

### 2.2. Treatment-Related Replenishment of CAFs 

Besides the resistant subpopulations, which survive anti-cancer therapy, CAFs are also replenished from other cell types [43]. The CAF-like dedifferentiation of carcinoma cells (via EMT) in experimental models and human tumors remains controversial. CAF-like phenotypes with EMT features have been detected both in breast and lung adenocarcinomas [60,61]. Single-cell RNA sequencing also identified CAFs with potential epithelial tumor origin. This EMT subclass can be distinguished from other CAFs by its scrapie-responsive protein 1 positivity and by its localization [62]. In contrast, EMT-based CAF differentiation was questioned in pharynx squamous cell- and colorectal adenocarcinoma xenografts [63]. In addition, bone-marrow-derived mesenchymal stem or stromal cells (BM-MSCs) can be recruited by the constantly evolving cancer microenvironment, and may also be important sources of CAFs. Active migration of BM-MSCs to tumor sites has been demonstrated in several studies [64,65]. BM-MSCs were detected in the tumor front and were shown to promote the survival of mammary and lung carcinoma cells upon exposure to paclitaxel or doxorubicin [66]. Furthermore, BM-MSCs contributed to the increase of CD133, octamer-binding transcription factor 4-positive and sex determining region Y-box 2-positive CSCs in a prostate cancer co-culture [64]. When cultured with cancer cells, they can differentiate to the CAF-like phenotype with tumor-supporting effect by sustained stromal-derived factor 1 expression [67]. Intriguingly, BM-MSCs share important similarities with CAFs, including (i) the expression of certain cell surface molecules (e.g., CD29, CD44, CD73, CD90, CD106 and CD117), (ii) the expression cytoskeleton and extracellular matrix proteins (e.g., vimentin, α-smooth muscle actin and nestin), as well as (iii) retention of pluripotency [68,69]. These data strongly suggest that BM-MSCs are not only progenitors of CAFs, but CAF-like cells may constitute a distinct subset of MSCs (i.e., CAF-MSCs).

Several studies described therapy-associated differentiation of fibroblasts to CAF-like cells, which suggests that fibroblasts in the normal tissue adjacent to the tumor can be important sources of CAFs. CAF-like differentiation of fibroblasts has been described after using sub-lethal doses of conventional chemotherapy in breast cancer models [48]. The treatment induced a metabolic switch in fibroblasts (a so-called catabolic tumor stroma phenotype) with increased secretion of interleukin-6 (IL-6) in co-cultures and resulted in activating stemness-related signaling (Hedgehog /GLI) in breast cancer cells [48]. Another study revealed increased transforming growth factor-β secretion after cisplatin treatment of non-small-cell lung cancer [70]. In turn, transforming growth factor-β-induced transition of fibroblasts to CAF-like cells with enhanced IL-6 secretion. Furthermore, CAF-conditioned media induced EMT, acquisition of stemness (i.e., CD133 expression) and cisplatin resistance in lung cancer cells [70]. MicroRNAs (miRs) as post-transcriptional regulators, may also take part in fibroblast differentiation to a chemoresistant CAF-like phenotype. For example, miR-27-transfected fibroblasts convert to CAFs with increased resistance to cisplatin [71]. RT may also cause biological and transcriptional changes in fibroblasts, which may directly affect stromal homeostasis [59]. The genes involved in this process, are related to DNA repair, cell cycle arrest, Wnt and IGF signaling, as well as to extracellular matrix remodeling [72]. RT can induce α-smooth muscle actin (fibroblast activation/CAF marker) expression consistent with myofibroblast differentiation in NIH-3T3 fibroblasts. Interestingly, RT did not affect α-smooth muscle actin, platelet-derived growth factor receptor-β and neuron-glial antigen 2 expression in NIH-3T3 cells in prostate cancer co-cultures, but it caused CAF-like differentiation and RT resistance in xenografts [73]. These results show that RT or chemotherapy induce normal fibroblasts to differentiate into resistant CAFs either directly or through forced paracrine signaling of the treated cancer cells.

### 2.3. Differential Cellular Behavior of Resting and Chemotherapy-Treated CAFs

It is also important to note that therapy-pressured CAFs may release cancer (minimal residual disease/CSCs)-supporting signals in contrast to non-treated CAFs. Elevated proportions of CD44^+^/CD24^low/−^ and aldehyde dehydrogenase-positive CSCs (the authors used the term tumor-initiating cells (TIC)) were induced in CAF cultures when treated with the maximum tolerated dose of paclitaxel and doxorubicin, compared to low-dose metronomic drug administration. In addition, maximum tolerated dose-treated patient-derived xenografts (breast cancer cells and CAFs) demonstrated an increased metastatic capacity compared to low-dose-treated mice. The latter also showed enhanced treatment response and extended survival [74]. Another study demonstrated that cisplatin treatment induces IL-11 release in CAFs, leading to chemo-resistance via the IL-11 receptor-α/signal transducer and activator of transcription 3 pathway in lung adenocarcinoma cells [75]. Moreover, CAFs treated with 5-fluorouracil, oxaliplatin and leucovorin promoted self-renewal of colorectal cancer-initiating cells by IL-17 signaling [58]. The tumorigenic capacity of CAFs was also increased by either a single ablative dose or a fractionated RT [59,76]. Elevated oncogenic signaling through fibroblast growth factor and insulin-like growth factor-1 release was also provoked in CAFs by gamma irradiation as compared to non-treated cells in colorectal and pancreatic cancers [77,78]. These data suggest that (i) the response to therapy of CAFs may be dose-dependent, and (ii) CAF-derived paracrine signals may play a fundamental role in the protection of residual cancer.

## 3. Extracellular Vesicle-Mediated Resistance at the Tumor Cell-CAF Interface

As described above, certain CAF subclones may survive different anti-tumor therapies. In parallel, the eliminated CAFs may be replaced by their progenitors. In addition, therapy-pressured CAFs are not passive elements of the niche, as they may support tumor growth more efficiently than non-treated CAFs or normal fibroblasts [26,77]. It is well known that CAF-released soluble factors and extracellular matrix components affect the sensitivity of cancer cells to therapy, and mediators released by tumors have an effect on CAF/fibroblast properties. 

### 3.1. Small EV-Induced Resistance in the CAF-Cancer Cell/CSC Axis

EVs can be classified based on their biogenesis. However, as their biogenetic origin often cannot be confirmed, a temporary nomenclature, based on, e.g., their biophysical properties such as size has been recently suggested for EVs [79]. Small EVs (sEVs, <200 nm in diameter) have intriguing and significant roles in the different phases of tumor progression and in the regulation of resistance mechanisms [79,80,81]. Certain reports described that both CAFs and cancer cells may dramatically increase release of sEVs with endosomal origin (i.e., exosomes, reviewed in [82]) caused by the effect of irradiation or chemotherapeutic agents [83,84,85]. The increased sEV release may be related to an increased DNA repair and p53 activation, which result in upregulation of tumor suppressor activated pathway-6 [86]. This suggests that the altered sEVs release may serve as an alarm signal in response to the therapeutic pressure. In general, cancer cell- or CAF-derived sEVs can affect clinically important properties of recipient cells [87,88,89]. These include the induced resistance against RT [90] and chemotherapeutic agents with different mechanisms of action (e.g., alkylating agents [90,91], antimetabolites [83,91,92] and mitotic inhibitors [87]). In the examples presented in Table 1, sEVs promote (i) signaling cascades (e.g., via the Wnt pathway or RNA-activated pattern recognition receptors) and (ii) regulation by miRs or long non-coding RNAs (lncRNAs). The majority of these studies emphasize the appearance of CSC phenotype (e.g., CD44, CD133, aldehyde dehydrogenase 1 and leucine-rich repeat-containing G protein-coupled receptor 5 (Lgr5) expression) and functions (e.g., chemoresistance, sphere formation, growth, and metastatic spread) as a result of EV-based communication between CAFs and cancer cells (Table 1). Nevertheless, the mechanism of therapy-induced CSC enrichment remains largely unclear. The increased percentage of CSC certainly cannot be explained solely by the death of sensitive cells (Figure 1), but it is rather due to the reprogramming of differentiated cancer cells. One of the main reprogramming factors is Wnt delivered by fibroblast/CAF-derived sEVs as described in colorectal carcinoma [92]. Wnt-β-catenin-TCF signaling drives the dedifferentiation of non-stem carcinoma cells to regain pluripotency and re-express Lgr5 and CD44 [93,94]. CAF-derived sEV miR-92a-3p caused an increased nuclear β-catenin expression in colorectal carcinoma cells with the appearance of CSC markers and several processes associated with EMT, such as the E-cadherin to N-cadherin switch [95]. Importantly, chemotherapy-treated CAF-released sEVs showed differential effects on the recipient cancer cells as compared to sEVs from untreated but resistant CAFs [83]. This supports the hypothesis that the cargo of therapy-induced EVs is different from that of the non-therapy-induced ones [96]. These results suggest that CAF-derived sEVs do not only mediate protective/supportive signals against the therapeutic stress, but they dynamically control the size of the CSC pool by the dedifferentiation process.

### 3.2. Medium-Sized EV Communication in Resistance Development of Tumor Cells and CAFs 

Medium-sized EVs (mEVs, earlier often designated as microvesicles) are directly produced by plasma membrane shedding and are characterized by heterogeneous size (~100 nm to 1 μm in diameter) [102]. mEVs are potent intercellular regulators with selectively loaded cargo, some of which appear to be associated with an unfavorable clinical outcome. Although the oncogenic effects of mEVs are less studied than those of sEVs, mEVs may have a bigger impact on the same processes as compared to sEVs. For example, CAF-derived mEVs show a strong influence on the proliferation of prostate cancer cells as compared to sEVs from the same CAF culture [103]. Stress factors, such as non-apoptotic doses of hypoxia or irradiation increase the secretion of tumor-derived mEVs in lung cancer cells. These fibroblasts showed increased secretion of soluble pro-angiopoietic and extracellular matrix-degrading factors (see Table 1 below for details). Functionally, by the activation of the mEV-fibroblast axis, tumor cells indirectly enhanced the angiogenic crosstalk in the tumor microenvironment [101]. Tumor-derived mEVs from prostate cancer cells decreased chemosensitivity in activated fibroblasts through phosphorylation of extracellular signal-regulated kinase 1/2. In addition, tumor-derived mEV treatment caused an increased mEV shedding from fibroblasts and this affected migration and invasion of highly metastatic PC3 cancer cells *in vitro* [100] (Table 1). Extracellular signal-regulated kinase 1/2 signaling was also involved in the metabolic switch of CAFs. This metabolic reprogramming provides energy-rich metabolites to adjacent cancer cells (i.e., reverse Warburg effect) and promotes survival of cancer cells in a nutrient-deprived environment. Oral squamous cell carcinoma-derived mEVs induce a metabolic switch from mitochondrial oxidation to glycolysis via transfer of phosphorylated extracellular signal-regulated kinase 1/2 and consequential degradation of caveolin 1 in normal fibroblasts. Tumor-derived mEV treatment increases lactate production in fibroblasts/CAFs, which consequently supports cancer progression *in vitro* and *in vivo* [104]. Caveolin 1-reduced, glycolytic CAFs protect estrogen receptor-positive MCF7 breast cancer cells against tamoxifen-induced apoptosis. Dasatinib, a tyrosine kinase inhibitor prevents the loss of caveolin 1 in CAFs and restores tamoxifen-sensitivity in MCF7 cells [105]. Summarizing these results, mEVs are multifunctional effectors in cancer cell-fibroblast/CAF interactions during the reaction to therapy and affect many cancer-related processes, such as metastatic potential, altered metabolism, and sensitivity of recipient cells. 

### 3.3. Apoptotic Cell-Derived EVs in the Development of Resistance, Relapse, CSC Phenotype and Metastasis

Several studies denote that the regenerative responses to cancer cell apoptosis paradoxically generate supporting signals and promote post-therapeutic relapse of residual tumors [106,107]. Apoptotic cell-derived EVs (apoEVs) are released because of the activation of the apoptosis effector machinery. ApoEVs carry a variety of bioactive molecules similarly to other vesicles. Furthermore, they contain caspase-modified autoantigens, nuclear remnants containing condensed chromatin, cellular organelles such as mitochondria and endoplasmic reticulum [108,109]. ApoEVs show a continuum in their size distribution (50–5000 nm in diameter) including the large apoptotic bodies (>1000 nm in diameter) [83]. Horizontal transfer of potentially oncogenic, genomic DNA through apoEVs may lead to the sustained transformation of recipient cells [108]. Tumor-derived apoEVs stimulate proliferation (termed compensatory proliferation or apoptosis-induced proliferation [108]) of surviving cancer cells in neighboring compartments [23]. The question can be raised whether these signals between apoptotic and surviving cancer cells lead to dedifferentiation to CSCs. An answer to this question is suggested by the observation that apoEVs induce proneural-to-mesenchymal transition (EMT-like phenotypic change) in glioblastoma cells with the appearance of CSC properties, such as expression of CD44 and aldehyde dehydrogenase 1A3, as well as resistance to therapy [23]. Emerging evidence suggests that apoptotic signals from dying cancer cells may promote a more supporting microenvironment called “onco-regenerative niche” [109]. For instance, mouse embryonic fibroblasts, deficient in p53 can reutilize H-ras^V12^, c-myc, and hygromycin resistance genes carried by apoEVs of cancer gene-transfected rat fibroblasts after their irradiation or nutrient-depletion to provide a selection advantage to the recipient cells [110]. Although, apoEV-based oncogenic gene transfer has been described between fibrosarcoma cells and p53-inactivated fibroblasts [111], only little is known about the significance of apoEV-based cancer cell-fibroblast/CAF interactions in therapy-induced stress reaction and post-therapeutic relapse. Starvation-induced, dying gastric carcinoma cell-derived apoEVs can promote co-invasion of the surviving cancer cells as small clusters with CAFs (i.e., CAF-led type invasion) [112]. To sum up these results, apoEVs may play a role in important processes of therapeutic and post-therapeutic tumor evolution, such as compensatory proliferation, horizontal oncogene transfer, and regulation of invasion. 

## 4. Conclusions

**Figure 2:**
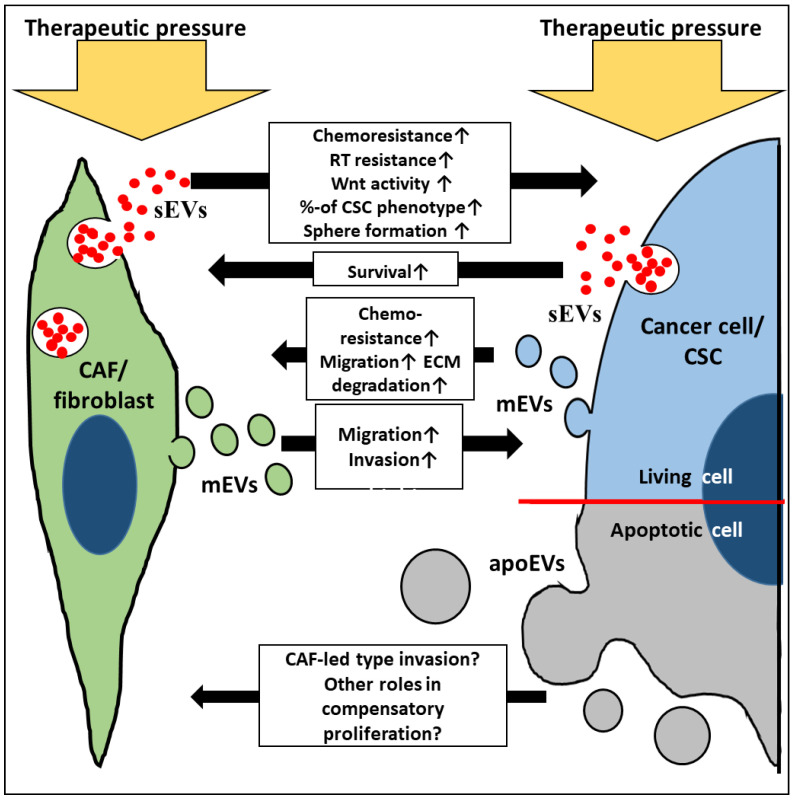
Simplified illustration of EV-based communication between therapeutic pressured fibroblasts/CAFs (green) and cancer cells/CSCs (blue and grey). apoEVs: apoptotic cell-derived EVs (grey circles); CSC: cancer stem cell; ECM: extracellular matrix; mEVs: medium-sized EVs (green circles: CAF/fibroblast origin, blue: cancer cell origin); RT: radiation therapy; sEVs: small EVs.

Anti-cancer therapies may act as double-edged swords. Besides their oncoreductive effects, they may also promote tumor evolution and progression though increasing the selection pressure on the surviving cells based on intratumoral heterogeneity. In cancer cells, treatment-related stress may be buffered by cells of the tumor microenvironment, especially CAFs. Accumulating data have shown that a therapy-induced, EV-based communication between fibroblasts/CAFs and the adjacent tumor mass may be critical in the cancer-stroma co-evolution and enrichment in the CSC populations. This leads to minimal residual disease, metastatic spread and tumor recurrence (Figure 2). The results overviewed from a variety of model systems, suggest that these treatment-induced interactions may occasionally endow tumors with a behavior, which is less favorable than what they had before the treatment. EVs can be crucial messengers here, their production is enhanced, and their molecular composition is modified upon treatment and tumor progression. Thus, EVs contribute to the intimate interplay between the cancer and the adjacent stroma. Therefore, it appears plausible that treatment-resistant surviving cancer cells may release a distinct EV profile. Furthermore, treatment-induced apoEVs may also carry nursing signals both towards cancer cells and stromal cells. All these features of EVs warrant high plasticity for the complex tumor tissue through activating diverse and simultaneous molecular pathways. Cancer cell and fibroblast subtypes, their therapy and time-dependent responses add further complexity to the EV-based interactions in the tumor-stroma co-evolution. Combined analysis of genetic and epigenetic features including tumor and EV heterogeneity may support the identification of novel therapeutic targets for more efficient cancers treatments.

## Figures and Tables

**Table 1:** Tumor cell-CAF EV-based crosstalk and its effect on chemotherapy and radiation therapy (RT).

Tumor Models (Cell Lines)	Origin of Extracellular Vesicles (and Used Terms)	Changes (↑↓) of Resistance- and CSC Properties as Well as Expression of Relevant Molecules and Activation of Pathways in the Recipient Cell Population.	Ref
Colorectal cancer: (SW620), xenograft	CAF-derived sEV(exosome)	Resistance to oxaliplatin and 5-FU↑. percentage of CD133^+^ CSCs↑, Wnt activity↑, in cancer cells	[91]
Colorectal cancer: (HT29, SW620), xenograft	Fibroblast (18Co) and CAF-derived sEVs (exosome)	Resistance to oxaliplatin and 5-FU↑. Wnt activity↑, cancer cell dedifferentiation to CSC↑, sphere formation ability↑	[92]
Colorectal cancer: (HCT116, SW480), xenograft	CAF-derived sEV (exosome)	Resistance to oxaliplatin↑, percentage of CSC marker positive cells↑, sphere formation ability↑	[97]
Colorectal cancer: (SW480, SW620, LOVO), xenograft	CAF-derived sEV (exosome)	Resistance to oxaliplatin and 5-FU↑. EMT and CSC markers↑, Wnt activity↑, mitochondrial apoptosis↓	[95]
Colorectal cancer: (HCT116, SW480), xenograft	CAF-derived sEV (exosome)	Resistance to oxaliplatin and 5-FU↑. colorectal cancer-associated lncRNA↑, Wnt activity↑	[88]
Pancreatic cancer: (PANC1, AsPC1), xenograft	CAF-derived sEV (exosome)	Gemcitabine resistance↑, Snail↑, miR-146a↑, proliferation↑, survival↑ in PaC cells	[83]
Ovarian cancer: (OVCA432, SKOV), xenograft	CAF-derived sEV (exosome)	Resistance to paclitaxel↑, miR-21↑, APAF1↓ in ovarian cancer cells	[87]
Ovarian cancer: (A2780, SKOV3), xenograft	CAF-derived sEV (exosome)	Resistance to cisplatin↑, CDKN1A↓ in ovarian cancer cells	[98]
Breast cancer: (MDA-MB-231), xenograft	CAF-derived sEV (exosome)	Resistance to RT and cisplatin↑, IRDS activation↑, RIG-I signaling↑, CD44^+^/CD24^low^ CSC subpopulation↑ in basal-like subtypes breast cancer cells	[90]
Head and neck cancer: (HN4, CAL27), xenograft	CAF-derived sEV (exosome)	Resistance to cisplatin↑, miR-196a↑, CDKN1B↓, ING5↓ proliferation↑, in recipient cancer cells	[99]
Breast cancer cells: (MDAMB23)	Tumor-derived sEV (exosome)	Paclitaxel treated tumor-derived sEVs promotes survival of fibroblasts	[89]
Prostate cancer: (PC3, LnCaP)	Tumor-derived mEV, fibroblast-derived mEV (microvesicle)	Tumor-derived mEV treated fibroblasts: chemosensitivity against actinomycin D↓, ERK1/2 phosphorylation↑, MMP9↑, migration↑. Fibroblast-derived mEV treated PC3 cells: migration↑, invasion ↑	[100]
Lung cancer: (LL-2, A549, HTB177), xenograft	Tumor-derived mEV (microvesicle)	Release of IL-1, -6, -8, -11↑, VEGF↑, LIF↑, OSM↑ and MMP9↑ in fibroblasts. Conditioned media of tumor-mEV treated fibroblasts: adhesion between LL-2 and HUVECs cells↑, metastatic potential of lung cancer cells↑	[101]

APAF1: apoptosis protease-activating factor-1; CDKN1B: cyclin dependent kinase inhibitor 1B; HUVECs: human umbilical vein endothelial cells; ERK1/2: extracellular signal-regulated kinase 1/2; ING5: inhibitor of growth 5; IRDS: interferon-related DNA damage resistance signature; LIF: leukemia inhibitory factor; MMP: matrix metalloproteinase; OSM: oncostatin M; RIG-I: retinoic acid-inducible gene-I-like receptor.
